# Sex differences in the impact of ozone on survival and alveolar macrophage function of mice after *Klebsiella pneumoniae *infection

**DOI:** 10.1186/1465-9921-9-24

**Published:** 2008-02-28

**Authors:** Anatoly N Mikerov, Xiaozhuang Gan, Todd M Umstead, Laura Miller, Vernon M Chinchilli, David S Phelps, Joanna Floros

**Affiliations:** 1 The Penn State Center for Host defense, Inflammation, and Lung Disease (CHILD) Research, Department of Pediatrics, The Pennsylvania State University College of Medicine, Hershey, PA 17033, USA; 2 Department of Cellular and Molecular Physiology, The Pennsylvania State University College of Medicine, Hershey, PA 17033, USA; 3 Department of Public Health Sciences, The Pennsylvania State University College of Medicine, Hershey, PA 17033, USA; 4 Department of Obstetrics and Gynecology, The Pennsylvania State University College of Medicine, Hershey, PA 17033, USA; 5 Capital Institute of Pediatrics, No.2 Yabao Road, Beijing 100020, China

## Abstract

**Background:**

Sex differences have been described in a number of pulmonary diseases. However, the impact of ozone exposure followed by pneumonia infection on sex-related survival and macrophage function have not been reported. The purpose of this study was to determine whether ozone exposure differentially affects: 1) survival of male and female mice infected with *Klebsiella pneumoniae*, and 2) the phagocytic ability of macrophages from these mice.

**Methods:**

Male and female C57BL/6 mice were exposed to O_3 _or to filtered air (FA) (control) and then infected intratracheally with *K. pneumoniae *bacteria. Survival was monitored over a 14-day period, and the ability of alveolar macrophages to phagocytize the pathogen *in vivo *was investigated after 1 h.

**Results:**

1) Both male and female mice exposed to O_3 _are significantly more susceptible to *K. pneumoniae *infection than mice treated with FA; 2) although females appeared to be more resistant to *K. pneumoniae *than males, O_3 _exposure significantly increased the susceptibility of females to *K. pneumoniae *infection to a greater degree than males; 3) alveolar macrophages from O_3_-exposed male and female mice have impaired phagocytic ability compared to macrophages from FA-exposed mice; and 4) the O_3_-dependent reduction in phagocytic ability is greater in female mice.

**Conclusion:**

O_3 _exposure reduces the ability of mice to survive *K. pneumoniae *infection and the reduced phagocytic ability of alveolar macrophages may be one of the contributing factors. Both events are significantly more pronounced in female mice following exposure to the environmental pollutant, ozone.

## Background

Lung disease is a major public health problem. Many patients have chronic respiratory ailments, including asthma and chronic obstructive pulmonary disease (COPD), while others are affected by acute diseases, such as pneumonia. Because of the widespread incidence of lung disease there have been a number of efforts to identify various risk factors. One factor that plays a role in many different diseases, from fetal life through old age, is sex. Males compared to females have been shown to have delayed lung maturation, a greater likelihood of suffering from neonatal respiratory distress syndrome after premature birth [1,2], and are more likely to be affected by idiopathic pulmonary fibrosis and COPD, although the changing demographics of smoking in recent years are giving rise to data that suggest increased incidence and severity of COPD in females [3]. Young males exhibit a greater incidence of asthma, although in adults, females are more affected. This changing picture suggests that the relationship between sex and lung disease is a complex one [3]. This complexity is evident when the incidence of pneumonia is examined. Females are less likely to develop most types of pneumonia and generally experience more favorable outcomes [4,5], with infection of mice by *Pseudomonas aeruginosa *and in cystic fibrosis patients being notable exceptions in which the male has a better outcome [6,7].

There are many different host defense processes that could potentially contribute to these sex differences in pneumonia. Phagocytosis is probably one of the most important and best understood, although little is known about whether sex has any influence on it. Phagocytosis is a complicated process that can involve a variety of cell surface receptors and may employ a variety of opsonins, such as surfactant protein-A or SP-A, to assist in the process [8,9]. Genetic variation, gene ablation, post-translational modification, and other perturbations of any of these molecules could potentially interfere with phagocytosis or some of its component processes, such as carbohydrate binding. Several examples of these perturbations are seen in the case of SP-A [10,11].

A number of factors may influence host defense. It has long been apparent that episodes of increased air pollution result in increased exacerbations or hospital admissions for a variety of respiratory ailments [12]. Although most studies have apparently not taken sex into account, there have been a few reports of sex differences in response to some pollutants [13]. The impact of O_3 _on pulmonary innate immunity, which has been reviewed recently [14], is an important one, as this has been associated with impaired host defense [15] and decreased survival in animals infected with *K. pneumoniae *[16]. The mechanisms by which various pollutants influence biological processes are incompletely understood, and it is likely that different pollutants (particulate matter, ozone, etc), involve different mechanisms [17].

Because many components of air pollution, ozone in particular, are potent oxidizing agents it is likely that these exert their effects by oxidatively modifying molecules such as proteins [18]. We have shown the ability of O_3 _to modify proteins [19], impair the cellular function of a macrophage-like cell line (THP-1) [20], and impair the function of surfactant protein-A (SP-A), a host defense molecule [21-23]. Recent *in vitro *studies showed that O_3 _exposure of SP-A can interfere with SP-A assisted phagocytosis of bacteria by macrophages)[24]. A decrease in alveolar macrophage phagocytic activity from mice exposed to O_3 _has been observed [25]. Moreover, O_3 _exposure of mice at the dose and time used in this study caused increases in certain inflammatory mediators, indices of tissue damage, and oxidative modification of proteins [26]. Under the conditions used in the published study inflammation was very limited and there was no mortality.

In the experiments described, we investigated the hypothesis that O_3 _exposure differentially affects survival and alveolar macrophage phagocytic function in male and female mice infected with *K. pneumoniae*. Towards this goal, we examined the impact of acute O_3 _exposure on the ability of mice to deal with a subsequent intratracheal challenge with *Klebsiella pneumoniae*. Survival rates and the phagocytic ability of alveolar macrophages from control and O_3_-exposed mice with pneumonia were investigated. To assess the impact of sex on these processes, both male and female mice were tested.

## Methods

### Animals

Male and female C57BL/6 mice (from Jackson Laboratory (Bar Harbor, ME)) were used at 8–12 weeks of age. The Penn State University Institutional Animal Care and Use Committee approved all procedures involving animals.

### Growing and preparation of bacteria

*Klebsiella pneumoniae *bacteria (ATCC 43816) were obtained from the American Tissue Culture Collection (Rockville, MD). These were inoculated into 50 ml of TSB in 250 ml flasks for 18 h at 37°C (stationary phase), with shaking at 120 rpm (Incubator Series 25, New Brunswick Scientific Co., Edison, NJ). The bacterial suspension was diluted in TSB to obtain an OD_660 _of 0.4; 200 μl of this was added to 50 ml of TSB for 3 h to reach mid-log phase of growth (OD_660 _~ 0.4, corresponding to ~2 × 10^8 ^CFU/ml), where bacteria are most virulent. For survival, the bacteria were placed on ice to stop growth and then serially diluted in PBS to obtain ~9 × 10^3 ^CFU/ml. For infection, 50 μl of suspension (~450 CFU/mouse) were used. For *in vivo *phagocytosis, the bacteria were sedimented at 2000 × g (20 min, 4°C), resuspended in PBS at ~2.4 × 10^8 ^CFU/ml, and 50 μl of this (~1.2 × 10^7 ^CFU/mouse) was used for infection of mice. The CFU/mouse doses stated above were found to be optimal for the respective studies in our preliminary experiments. CFU per ml values were estimated based on OD_660 _of the bacterial suspension. An aliquot was also spread on TSA plates to confirm CFU estimates. The bacterial suspension was then used right away.

### Exposure of mice to O_3_

Mice were exposed to O_3 _(2 ppm for 3 h) or to FA (control) at the same time in different chambers as described [26]. This dose/duration was chosen in our preliminary work as being optimal for further investigations [26]. Mice were infected immediately after exposure. For survival, sixteen experiments, each involved 5 mice exposed to O_3 _or to FA; for *in vivo *phagocytosis, ten experiments, each involved 3 mice exposed to O_3 _or to FA.

### Infection of mice with *K. pneumoniae *for survival analysis

**Figure 1:**
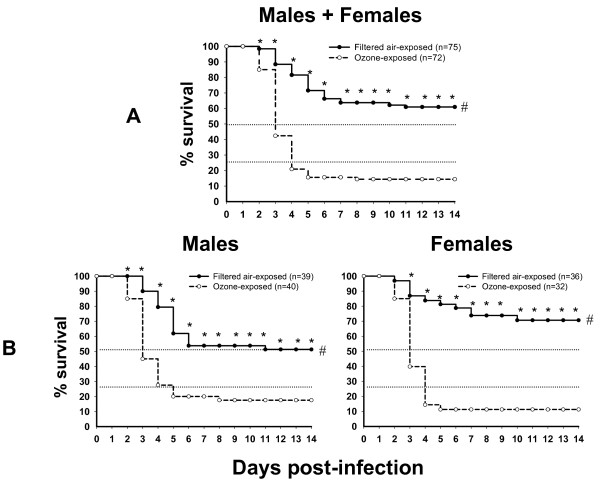
**Effect of ozone exposure on survival of mice with *K. pneumoniae *infection; sex differences**. Male and female mice were exposed to O_3 _(2 ppm for 3 h) or to FA (control) and then infected intratracheally with ~450 CFU of *K. pneumoniae *bacteria. Panel A depicts the results for all mice regardless of sex, and Panel B shows the results for males and females plotted separately. Percent surviving is shown up to 14 days. Differences between survival rates of FA- and O_3_-exposed mice were analyzed with log-rank test (cumulative survival for all 14 days) and with either Chi-Square test (Panel A) or with Fisher's Exact test (Panel B) (daily survival). Differences were considered significant if *p *< 0.05. Significant differences by log-rank test (#) or by Chi-square or Fisher's Exact test (*) (daily survival) are indicated.

**Figure 2:**
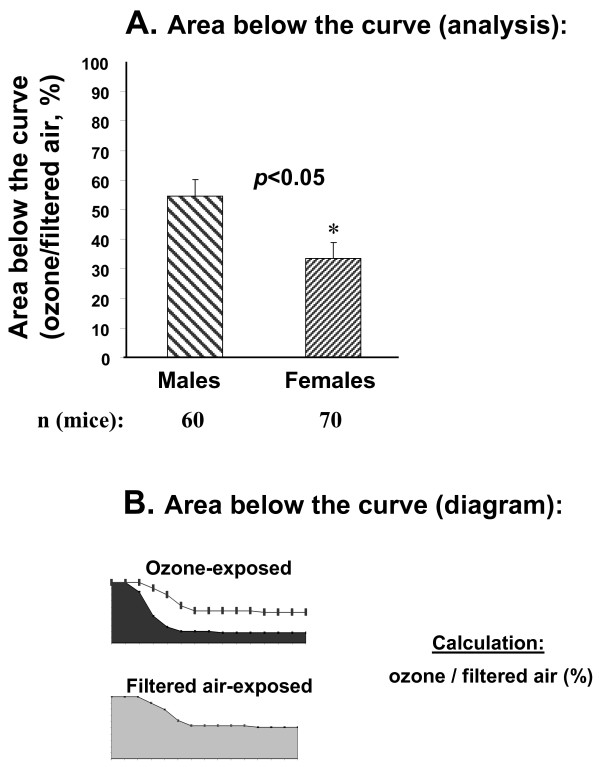
**Comparison of "cumulative" survival rates of males and females after ozone-exposure followed by *K. pneumoniae *infection**. Experimental design was described in the legend for Figure 1, and in the Methods section. Panel A: Data (in percent of control – FA) for differences in survival between males and females. Panel B: Graphic representation of area below the curve (O_3 _and FA depicted by black and grey filled areas, respectively) of data shown in Panel A. The experiments from which these data are derived are in Panel B of Figure 1. The area below the curve was calculated with Sigma Plot 10.0 Software. O_3 _(top) and FA (bottom) curves. The resulting ratios (O_3_/FA times 100%) for males vs. females were compared with a t-test. Results were considered significant when p < 0.05, and are indicated with an asterisk, *.

Animals were anesthetized with an intramuscular injection of a mixture of Ketamine HCl (Ketaset, Fort Dodge Animal Health, IO) and Xylazine (XYLA-JECT, Phoenix Pharmacueticals Inc., St. Joseph, MO). The trachea was surgically exposed and ~450 CFU/mouse were inoculated intratracheally in 50 μl of PBS. Skin incision was closed with 7 mm wound clips. Deaths during the first 12 h post-infection period were considered to be due to surgical procedure rather than infection and those mice were excluded from study. Sixteen independent experiments (8 males and 8 females) were conducted. Each experiment consisted of 10 mice (5 exposed to FA or to O_3_). The mice were monitored for survival for 14 days. The total number of mice used for Figure 1 was 147 mice (75 FA-exposed [39 males+36 females]), and 72 O_3_-exposed [40 males + 32 females]). In cases (n = 14) where mice were moribund with no chance of recovery, these were euthanized to prevent unnecessary suffering according to Penn State University Institutional Animal Care and Use Committee recommendations and are included with the natural deaths. For the analysis shown in Figure 2, 130 mice were used out of the total 147 mice. Of these, 60 were males (6 independent experiments out 8) and 70 females (7 independent experiments out 8). Ratios from two experiments using males and from one with females were eliminated from analysis because the values measured were more than 2 standard deviations from the mean.

### Infection of mice with *K. pneumoniae *for *in vivo *phagocytosis analysis

Experimental design of *in vivo *phagocytosis was the same as described above for the survival study with the exception of the bacterial dose (~1.2 × 10^7 ^CFU/mouse in 50 μl of PBS). Five independent experiments were performed, each involved 3 mice exposed to O_3 _or to FA. To harvest alveolar macrophages, mouse lungs were subjected to bronchoalveolar lavage (BAL) (3× with 0.5 ml of 0.9% NaCl) at 1 h after infection, and kept on ice. If a mouse died within 1 h after infection, the lung was not lavaged and this mouse was excluded from analysis. Alveolar macrophages were prepared as described previously [11] and applied to slides using a cytocentrifuge. The slides were stained using the Hema-3 Stain Kit for analysis by light microscopy. The phagocytic index was calculated as described: the percent of bacteria-positive alveolar macrophages (macrophages that phagocytized at least one bacterium) × average number of bacteria per bacteria-positive alveolar macrophages [27]. The actual values calculated for the phagocytic index were used for this analysis, rather than percentages or normalized values.

### Statistics

Survival data were analyzed with log-rank test (cumulative survival, for entire 14 day period), with either a Chi-Square test or with Fisher's Exact test (daily survival). Proportions of animals surviving under different conditions were compared with a Z-test. Areas below the survival curves and *in vivo *phagocytosis data were analyzed with simple t-test. In each of these cases the analysis used is given in the respective Figure legends. Results were considered statistically significant when *p *< 0.05.

## Results

In this work we used the term "sex differences" rather than "gender differences" to correctly reflect the nature of results described in this study. Recent review articles have clearly defined in which case the term "sex" or "gender" is appropriate [3,28]. "Sex differences" are commonly referred to as differences generated by genetic and biological factors, whereas "gender differences" are associated not only with physiological differences, but also include the social, economic, and cultural factors that are applicable to human subjects only.

### Effect of O_3 _exposure on survival of mice after *K. pneumoniae *infection

To test our hypothesis that O_3 _exposure differentially affects survival of male and female mice after *K. pneumoniae *infection, we first exposed mice to O_3 _or to FA (used as a control) and then infected them with *K. pneumoniae *bacteria. We then evaluated the data obtained in several ways to compare survival: a) in O_3_-exposed vs. FA-exposed mice; and b) in males vs. females. The entire survival study involved 147 mice.

In preliminary experiments, a bacterial dose of ~450 CFU per mouse was found to be optimal to study differences in survival rates. At this dose there was about 50% mortality rate (LD_50_) in unexposed male mice over a 14-day observation period. This dose approximates that used in published reports where infection with *K. pneumoniae *was examined [29]. We also confirmed, prior to experimentation, that O_3 _and FA exposure had no adverse effect on the survival of mice inoculated with PBS only (no bacteria). Both the cumulative survival (over the entire 14 day period) as well as the daily survival rates were analyzed. To facilitate comparisons between different groups of mice, we placed dotted reference lines across the graphs at 50% and 25% survival (Figure 1).

#### Survival rates regardless of sex

Figure 1A demonstrates the comparison of survival rates of O_3_- vs. FA-exposed mice. Analysis of cumulative survival with log-rank test revealed that survival rates of mice exposed to O_3 _are significantly lower than those exposed to FA. When we compared (with Chi-square test) the daily survival rates of O_3_- and FA-exposed mice after *K. pneumoniae *infection, we found that survival of O_3_-exposed mice was significantly lower (p < 0.05) than that of FA-exposed mice on days 2–14. On day 14, the final day of observation, the survival rates (%) were 60.9% ± 5.6 and 14.4% ± 3.1 for FA- and O_3_-exposed mice, respectively. We concluded that mice exposed to O_3 _are significantly more susceptible to *K. pneumoniae *infection than mice exposed to FA, and that this susceptibility reaches significance from day 2 onwards following infection.

#### Survival rates in males and females

Figure 1B demonstrates the impact of sex on the cumulative and daily survival rates of O_3_- vs. FA-exposed mice and clearly demonstrates that the distance between FA and O_3 _survival curves, depicting the shift from control conditions (FA) to O_3 _exposure conditions, is much greater in females compared to males.

##### i) Cumulative survival rates

The cumulative survival analysis revealed that the survival of O_3_-exposed mice was significantly lower than that of FA-exposed in both males and females. On the final day of observation (day 14) the survival rates were 51.2% ± 6.4 and 17.5% ± 4.5 for males and 70.6% ± 8.2 and 11.2% ± 4.3 for females, for FA- and O_3_-exposed mice, respectively. Although survival of FA-exposed males was lower than that of females and survival of O_3_-exposed males was higher than that of females (see Figure 1B), the differences between survival rates of FA-exposed males and females or between O_3_-exposed males and females were not statistically significant.

However, on day 14, relative to control conditions (FA-exposed mice), the survival of O_3_-exposed male mice decreased 2.9 times (51.2%: 17.5%); whereas the survival for females decreased 6.3 times (70.6%: 11.2%). Furthermore, we used an alternative method to assess "cumulative" survival and to compare sex differences in survival. For this, the physical area below the curve of O_3_-exposed to FA-exposed mice was calculated (Figure 2). This method takes into account differences throughout the time course. This type of analysis demonstrated that female mice are significantly more susceptible to pneumonia after O_3 _exposure than males. The values (in percent) obtained from the area below the curve of the surviving males were 54.7% ± 6.5 (O_3_: FA = relative areas of [520 ± 52]: [954 ± 32]) and 33.4% ± 5.7 (O_3_: FA = relative areas of [372 ± 53]: [1163 ± 80]) for females. Thus, the risk of dying from pneumonia after O_3 _exposure is nearly twice as high for female mice as it is for male mice.

##### ii) Daily survival rates

**Figure 3:**
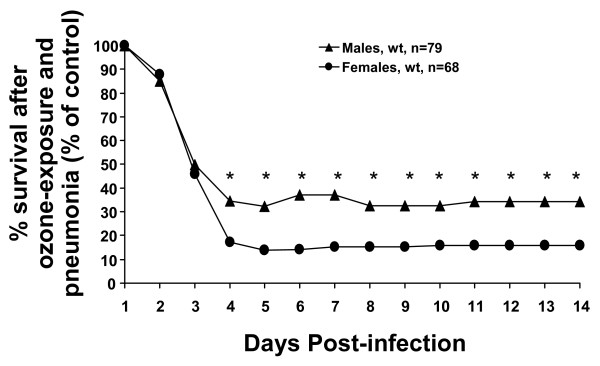
**Comparison of daily survival rates of males and females after ozone-exposure followed by *K. pneumoniae *infection**. Experimental design was performed as described in the legend for Figure 1 and the Methods section. Data in Figure 3 are presented as percent survival. Survival of mice in the control male or female group (FA-exposed) was set as 100%, and survival in the corresponding O_3 _group was calculated as a percent of the control group (O_3_/FA × 100%). For statistical analysis, proportions (O_3_/FA absolute survival rates) for males and females were compared daily with Z-test. Significance between male and female mice was noted if p < 0.05 (indicated with an asterisk, *).

As shown in Figure 1B, the daily survival rates for O_3_-exposed mice were significantly lower than those for FA-exposed animals on days 2–14 for males and 3–14 for females, respectively. To assess differences in daily survival between males and females with regard to their ability to survive *K. pneumoniae *infection, the proportions of O_3_-exposed males or females surviving to FA-exposed males or females surviving for each day (1–14) were calculated and shown in Figure 3. Females were significantly more susceptible and at higher risk of dying from pneumonia after O_3 _exposure than males on days 4–14.

### *In vivo *phagocytosis by alveolar macrophages from O_3_- or FA-exposed mice

**Figure 4:**
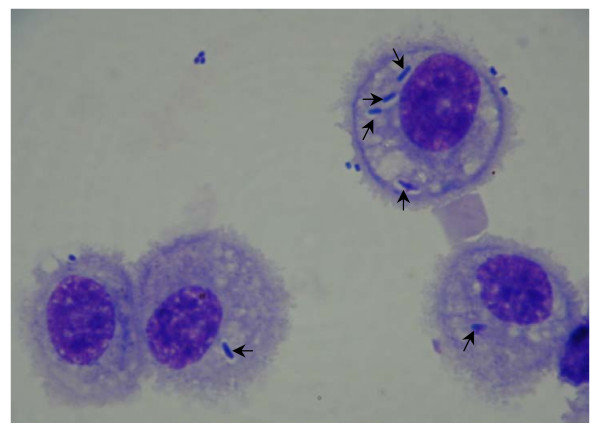
**Microscopic view of mouse alveolar macrophages with *K. pneumonia *bacteria phagocytized *in vivo *(in the lung)**. The arrows show bacteria phagocytized by macrophages. These were counted for the analysis shown in Figure 5 (×1000; oil immersion), as described in Methods.

**Figure 5:**
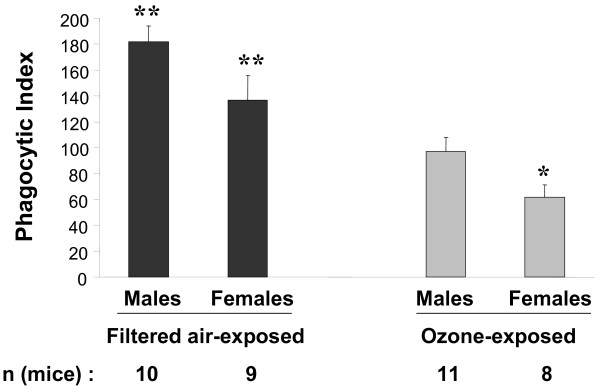
***In vivo *phagocytosis of *K. pneumoniae *by alveolar macrophages from mice, exposed to ozone**. Experimental design was as described in the legend for Figure 1 and the Methods section except that a dose of ~1.2 × 10^7 ^CFU/mouse of *K. pneumoniae *was used, and the mouse lungs were lavaged 1 h post-infection, as described in Methods. Alveolar macrophages were prepared, applied to slides by cytocentrifugation and stained for phagocytosis assessment by light microscopy (see Methods). Results were considered to be significant if p < 0.05 with t-test. (*) significant differences between males and females of the same group (i.e. O_3 _or FA); (**) significant differences between FA and O_3 _groups of mice of the same sex. The number of independent experiments was 5 and the number (n) of mice in each group that survived after the procedure is shown in the figure.

Figure 4 shows a representative microscopic view of *K. pneumonia *bacteria phagocytized by mouse alveolar macrophages *in vivo*. The phagocytic index was calculated as described in Methods. The analysis revealed that the phagocytic indices of alveolar macrophages isolated from FA-exposed female mice appeared to exhibit lower activity than those from male mice (181.6 ± 12.2 for males and 136.9 ± 19.0 for females) (Figure 5), although the differences did not reach statistical significance (*p *= 0.06). The phagocytic indices from O_3_-exposed mice differed (*p *= 0.03) between males and females (96.9 ± 11.0 for males and 61.6 ± 9.6 for females). The phagocytic index for females was about 64% of that for males (*p *< 0.05). Thus, the data in Figure 5 show that although O_3 _exposure causes a significant reduction in the phagocytic index of alveolar macrophages in both sexes, this reduction is greater in females. When the proportions of the phagocytic indices of alveolar macrophages from FA- to O_3_-exposed male or female mice were determined, the O_3_-induced decrease in the phagocytic index for males was about 1.9-fold (182 : 97) and for females was 2.2-fold (137 : 62).

## Discussion

Several factors have been identified as risk factors in lung disease. Sex is one of the factors that may differentially modulate lung disease susceptibility. In this report, we investigated the hypothesis that O_3 _exposure differentially affects the survival of male and female mice with pneumonia. Male and female mice were exposed to either FA (control) or O_3 _and then infected with pathogenic bacteria immediately after exposure. We studied their cumulative and daily survival over a period of 14 days and assessed the phagocytic capability of alveolar macrophages isolated from O_3_- or FA-exposed and infected mice. The results showed that: 1) mice, regardless of sex, exhibit reduced survival if exposed to O_3 _prior to infection; 2) while female mice appear to be more resistant to infection with the pathogenic microorganism, *K. pneumoniae*, than male mice, they are significantly more sensitive to an injurious environmental stimulus (in this case O_3 _exposure) and exhibit a greater reduction in their survival rate than male mice; 3) alveolar macrophages isolated from mice exposed to O_3 _prior to infection exhibit reduced phagocytic activity compared to those that were not exposed to O_3_; and 4) alveolar macrophages from O_3_-exposed female mice exhibited a greater reduction in phagocytic activity compared to those from male mice. The findings clearly document the impact of a prior environmental insult to the lung on subsequent infection, sex-related differences in the susceptibility to lung infection, and the role of the alveolar macrophages in the process. Furthermore, the data underscore the importance of considering sex in studies where the impact of environmental pollution on lung infection is being investigated.

Although both males and females appear to have similar respiratory requirements, numerous studies have documented sexual dimorphism in lung function and lung disease pathogenesis throughout fetal developmental and postnatal life. These have recently been reviewed [30]. A number of clinical and animal studies have demonstrated sexual dimorphism in the incidence and severity of pneumonia [5,31] and other lung ailments [3]. Data from human clinical studies typically show that females are less susceptible to pneumonia and have a better outcome [5,32]. These include studies of patients who have contracted pneumonia after undergoing major trauma [5,33], a large study of nosocomial pneumonia [34], and studies of community-acquired pneumonia [4,35]. Moreover, elderly blacks are shown to be more susceptible to pneumonia caused by *K. pneumoniae *than whites of the same age. However, in both, black and white study groups, females were less susceptible to pneumonia, indicating that race is not a factor in this sex difference [32]. Collectively, the published findings from human clinical studies are largely consistent with our observations that FA-exposed female mice appear to be less susceptible to infection with *K. pneumoniae *than FA-exposed male mice.

In contrast to pneumonia, when the injurious agent falls in the category of environmental pollutants, the male disadvantage or female advantage appears to reverse. Elderly females have been shown to have a more pronounced response to ambient air pollution than males [13] and some experimental studies have shown that the lungs of female mice sustain greater damage after naphthalene exposure than those of males [36]. Our data showing increased susceptibility to pneumonia in female mice after O_3 _exposure are consistent with the studies reporting increased vulnerability to environmental insults by females. Based on the present findings and those from published reports, we speculate that environmental pollutants, whether from air pollution or cigarette smoke, have a negative and perhaps a more deleterious impact on females than males. Therefore, taken together, the available information points to a need for gender-based studies when the impact of environmental insults on lung health is being considered.

Previous work with the *K. pneumoniae *model, used in the present study, showed that normal clearance of *K. pneumoniae *is primarily dependent on macrophages [37] and that several days of nonlethal hyperoxia can severely compromise its clearance [29]. As with the hyperoxia study, the O_3 _exposure in the present study severely compromised the ability of the mice to clear the bacteria as assessed by the observed decrease in survival and phagocytic ability of alveolar macrophages. However, the fact that the effects described here were seen after a single acute exposure to O_3_, rather than the several day exposure to hyperoxia previously reported indicates that different or overlapping mechanisms may be operative, although in both cases it is likely that oxidative mechanisms are involved.

Exposure of mice to O_3 _resulted previously in increased levels of protein oxidation in BAL at 4 hr or more after O_3 _exposure [26]. It is tempting to speculate that the increases in protein oxidation observed in the Haque study are linked to deficits in bacterial clearance in the present study and result in increased mortality after O_3 _exposure followed by the infection. However, even though no increases in total protein oxidation were reported immediately after the termination of O_3 _exposure, a time frame similar to that used in the present study, specific proteins critical for macrophage phagocytic function, such as SP-A, may be preferentially oxidized. In fact, the oxidation level of SP-A was increased right after O_3 _exposure [26]. Of interest, O_3 _exposure of a macrophage-like human cell line caused a decrease in its responsiveness to a subsequent stimulus [20]. This *in vitro *finding of decreased responsiveness is consistent with our present *in vivo *observations and published findings [25] where the ability of alveolar macrophages to enhance phagocytosis was decreased in mice exposed to O_3_.

Understanding how the behavior of effector cells, especially alveolar macrophages, are influenced under specified conditions is an important first step. How single or multiple treatment agents can differentially alter phenotype between males and females is exemplified by the observation that spontaneous production of nitric oxide in alveolar macrophages from female mice increases after LPS treatment alone [38], as does neutrophil phagocytosis and cytokine production (TNF-α) [39]. However, higher amounts of nitric oxide are produced by alveolar macrophages from male mice following LPS treatment and interferon-γ stimulation [38]. While these processes may play a role in host defense against *K. pneumoniae*, multiple factors including several cytokines [40], lysozyme [41], epithelial cell ICAM-1 [42], and other factors such as apolipoprotein E [43] are all involved in combating *K. pneumoniae*. None of these, with the exception of one or two of the cytokines have been examined for evidence of sexual dimorphism.

In the current investigation, O_3_-exposure had a more negative impact on the phagocytic activity of macrophages from female mice. Alveolar macrophages isolated only 1 hr after infection from female mice, exhibited lower activity than those from male mice; this is consistent with the results of the survival study and indicates that alveolar macrophage-mediated mechanisms contribute to the observed sex-based response to *K. pneumoniae *infection. Although macrophages from female FA-exposed mice appeared to have a lower (albeit not significant) activity than macrophages from male mice, this observation indicates that other factors contribute to host defense against *K. pneumoniae *and the higher survival rate in female mice exposed to FA. Among these may be antibodies, which are expressed at higher levels in females than in males [44]. Furthermore, the rapidity of this event would tend to suggest a direct effect of O_3 _exposure on the phagocytic process, which in turn may contribute to differences in survival.

Deciphering the overall basis of the observed sex differences in the susceptibility to the lung infection after exposure to ozone may be very complex and may also involve many processes other than phagocytosis, including those that are dependent on the continuing presence of circulating sex hormones, those that are the result of sexual differentiation and may not require the continued presence of hormones, and oxidative as well as other mechanisms that may be involved in lung health. Using a model of post-trauma sepsis it was reported that there is increased damage to the lungs of males versus females, a difference that is abrogated if estrogen and progesterone are given to males [45]. With regards to susceptibility to infection, one study showed that female mice are more susceptible to infection with *Pseudomonas aeruginosa *than males [6], while another concluded that male mice are more susceptible than females to infection with *Mycoplasma pulmonis *[31]. The latter difference was abolished when the males were castrated, suggesting that male sex hormones were responsible. The reasons behind the varying differences between males and females are not known, but may be partly related to specific organisms. A recent study [46] demonstrated that male sex hormones promote reflex airway responsiveness to vagally-mediated cholinergic stimulation that may be important in the regulation of airway tone in the normal and diseased lung. Sexual dimorphism has been also documented in the anatomy of the distal lung and airways [47,48]. These differences may also affect susceptibility to infection, the efficiency of host defense functions, and could also influence the penetration of air pollutants to the distal lung and the response to them.

In the present study, we demonstrated that although male mice appear to be more susceptible to infection with *K. pneumoniae *than females, exposure to O_3 _reverses this pattern and renders the females significantly more susceptible, as assessed by the survival studies. We also showed that the *in vivo *phagocytic function of alveolar macrophages from female mice exposed to O_3 _prior to infection is significantly lower than those from male mice and therefore macrophage-mediated mechanisms may constitute one of the contributing processes to the observed sex-based differences. Furthermore, these findings in addition to pointing to the complexities of lung disease in response to different types of insults, allow us to speculate that the effect of air pollution, and particularly of ozone, on the immune lung status of patients has a significantly higher negative impact in women than in men. The latter highlights the need to independently assess each sex in experimental models, especially when the role of environmental pollutants in lung health is being investigated. Should these observations translate (in subsequent studies) to comparable findings in the human population, it may warrant a rethinking of what constitutes a sensitive population as ozone exposure standards are being set.

## Conclusion

Mice exposed to ozone are significantly more susceptible to *K. pneumoniae *infection than mice exposed to filtered air, and ozone exposure decreases the survival of female mice after *K. pneumoniae *infection significantly more than it does in males. The ability of alveolar macrophages to phagocytize *K. pneumoniae *bacteria is also reduced in response to ozone, and the phagocytic ability of macrophages from females is affected more than macrophages from males. The data indicate a greater susceptibility to respiratory infection for females compared to males following exposure to the environmental pollutant, ozone.

## Competing interests

The author(s) declare that they have no competing interests.

## Authors' contributions

ANM set up the infection model, the *in vivo *phagocytosis model, planned and carried out all experiments, analyzed and interpreted data, and wrote the manuscript. XG participated in experiments of survival study. TMU contributed in all aspects of setting up the ozone exposure model. LM participated in the *in vivo *phagocytosis experiments. VMC participated in statistical analysis of data for the survival study. DSP contributed in the planning of experiments, interpretation of data, and writing of the manuscript. JF contributed in the design of the project, analysis and interpretation of data, and the writing of the manuscript. All authors read and approved the final manuscript.
